# Validation of the Perceived Stigmatization Questionnaire for Brazilian adult burn patients

**DOI:** 10.1371/journal.pone.0190747

**Published:** 2018-01-30

**Authors:** Noélle de Oliveira Freitas, Carlos García Forero, Marina Paes Caltran, Jordi Alonso, Rosana A. Spadoti Dantas, Monica Sarto Piccolo, Jayme Adriano Farina, John W. Lawrence, Lidia A. Rossi

**Affiliations:** 1 Inter-institutions Doctoral Program in Nursing, University of São Paulo at Ribeirão Preto College of Nursing, Ribeirão Preto, Brazil; 2 Health Services Research Unit, IMIM Hospital del Mar Medical Research Institute, Barcelona, Spain; 3 University of São Paulo at Ribeirão Preto College of Nursing, Ribeirão Preto, Brazil; 4 CIBER Epidemiología y Salud Pública, (CIBERESP), Madrid, Spain; 5 Department of Experimental and Health Sciences, Universitat Pompeu Fabra, (UPF), Barcelona, Spain; 6 General and Specialized Nursing Department, University of São Paulo at Ribeirão Preto College of Nursing, Ribeirão Preto, Brazil; 7 Emergency Department for Burns at the Hospital Nelson Piccolo, Goiânia, Brazil; 8 University of São Paulo, Ribeirão Preto Medical School, Head of the Division of Plastic Surgery and the Burn Unit at Hospital das Clínicas, Ribeirão Preto, Brazil; 9 The College of Staten Island, City University of New York, Staten Island, New York, United States of America; Hong Kong Polytechnic University, HONG KONG

## Abstract

Currently, there is no questionnaire to assess perceived stigmatization among people with visible differences in Brazil. The Perceived Stigmatization Questionnaire (PSQ), developed in the United States, is a valid instrument to assess the perception of stigmatizing behaviours among burn survivors. The objective of this cross-sectional and multicentre study was to assess the factor structure, reliability and validity of the Brazilian Portuguese version of the PSQ in burn patients. A Brazilian version of the 21-item PSQ was answered by 240 adult burn patients, undergoing rehabilitation in two burns units in Brazil. We tested its construct validity by correlating PSQ scores with depression (Beck Depression Index-BDI) and self-esteem (Rosenberg Self-Esteem Scale-RSE), as well as with two domains of the Revised Burn Specific Health Scale—BSHS-R: affect and body image, and interpersonal relationships. We used Confirmatory Item Factor Analysis (CIFA) to test whether the data fit a measurement model involving a three-factor structure (absence of friendly behaviour; confusing/staring behaviour; and hostile behaviour). We conducted Exploratory Factor Analyses (EFA) of the subscale in a 50% random sample of individuals (training split), treating items as ordinal categorical using unweighted least squares estimation. To assess discriminant validity of the Brazilian version of the PSQ we correlated PSQ scores with known groups (sex, total body surface area burned, and visibility of the scars) and assessed its reliability by means of Cronbach's alpha and using test-retest. Goodness-of-fit indices for confirmatory factor analysis were satisfactory for the PSQ, but not for the hostile behaviour subscale, which was modified to improve fit by eliminating 3 items. Cronbach’s alphas for the PSQ refined version (PSQ-R) ranged from 0.65 to 0.88, with test-retest reliability 0.87 for the total score. The PSQ-R scores correlated strongly with depression (0.63; *p* < 0.001), self-esteem (-0.57; *p* < 0.001), body image (-0.63; *p* < 0.001), and interpersonal relationships (-0.55; *p* < 0.001). PSQ-R total scores were significantly lower for patients with visible scars (effect size = 0.51, *p* = 0.029). The PSQ-R showed reliability and validity comparable to the original version. However, the cross-cultural structure of the subscale “hostile behaviour” and sensitivity to change of the PSQ should be further evaluated.

## Introduction

That some people may develop psychopathology due to the visible result of a physical injury is well known [1]. The consequences can be aggravated by stigmatization, during social interactions, when other people evaluate negatively the person with a visible difference [2], resulting in social or material losses or both. People with visible differences, such as those resulting from a burn injury, frequently report perceiving stigmatizing behaviour in others, such as staring, comments and questions [3], bullying, avoidance behaviour, teasing and external pressure to change their appearance [4]. Such stigmatization behaviours may lead these individuals to experience further feelings of social discomfort, social anxiety, distress, poor body image and low self-esteem, resulting in social isolation [5,6].

Instruments for measuring stigmatization have been developed for a variety of mental disorders [7], obesity [8], HIV/AIDS and tuberculosis [9], as well as for caregivers [10,11] and epileptics [12]. However, a recent systematic review of patient-reported outcome measures (PROMs) administered to burn survivors [13] has found that the Perceived Stigmatization Questionnaire (PSQ) and the Social Comfort Questionnaire (SCQ) are the only two generic PROMs which have been psychometrically evaluated among both child (from 8 to 18 years old) and adult burn survivors [4,14]. The PSQ is a 21-item questionnaire designed to measure perceived stigmatization among people with visible differences. It was developed in the US and it has shown good validity among both paediatric and adult burn survivors [4,14]. We have not found any methodological study reporting cross-cultural adaptation of the PSQ into any other language. Although there is one publication describing the use of the PSQ in German children and adolescents with congenital and acquired differences [15] we could not find any publication reporting the psychometric properties of the PSQ in German.

In a previous study [16], we described the translation and cultural adaptation process of the PSQ for Brazilian-Portuguese adult survivors of burns and ensured its semantic, idiomatic, experiential and conceptual equivalence, following the steps recommended in the literature for translation and cross-cultural adaptation [17,18]. We tested the internal consistency of the Brazilian version of the PSQ in a sample of 30 patients and the Cronbach’s alpha was 0.87 for the total score [16]. In the present study, we describe the testing of the psychometric properties of the PSQ Brazilian version (BR-PSQ) in a larger sample than that of the previous study [16]. Thus, in this study, we assessed the factor structure, reliability and validity of the Brazilian version of the PSQ in Brazilian burn patients.

We hypothesized that perceived stigmatization would be at least moderately to strongly positively correlated with depression; at least moderately to strongly negatively correlated with interpersonal relationships; at least moderately to strongly negatively correlated with affect and body image; and at least moderately to strongly negatively correlated with self-esteem. In addition, we hypothesized that the Brazilian version of the PSQ would show a three-factor model structure and we explored the relationship between the Brazilian version of PSQ and the following factors: scar visibility, body surface area burned- BSA (BSA <20% vs ≥20%), and gender.

## Methods

### Design

This cross-sectional, multicentre study was conducted with outpatients from two different sources: the burns unit at the University Hospital affiliated with the University of São Paulo School of Medicine, in Ribeirão Preto, state of São Paulo and the Emergency Department for Burns at the Hospital *Nelson Piccolo* in Goiânia, state of Goiás, Brazil.

### Participants

Patients were invited to participate after their appointment at the burns unit. Inclusion criteria were: Brazilian burn patients older than 18 years of age, who had been discharged between two and 12 months ago and were awaiting reconstructive surgery, and outpatients being followed by the Burns Unit. Burn patients who had not required hospitalization were also included. Patients were excluded if they had any prior psychiatric diagnosis (registered in patients charts), or cognitive difficulties (assessed by the ability to indicate the address where they lived, day of week and month, age or date of birth) as well as those whose burns were the result of a suicide attempt.

We established sample size following the generic guideline of 5–10 subjects per item of the instrument [19]. The PSQ has 21 items and the final sample included 240 burn patients.

### Procedures and ethics statement

The study proposal was approved by the Ethics Committee from the University of São Paulo at Ribeirão Preto College of Nursing (protocol number 04618612.5.0000.5393). All patients received information about the study and signed an informed consent.

Data collection was conducted from 24^th^ January 2013 to 18^th^ December 2014. After their appointment at the burns unit, patients participated in a face-to-face interview. We administered the BR-PSQ together with other questionnaires. In addition, participants were asked for sociodemographic details and burn characteristics. We assessed BSA and number of surgical operations by reviewing the participant’s chart.

A subsample of 30 patients was re-administered the BR-PSQ 2–4 weeks later, in order to assess test-retest reliability.

### Measures

#### Perceived Stigmatization Questionnaire (PSQ)

The BR-PSQ contains 21 items divided into three subscales: subscale 1—absence of friendly behaviours (items 1, 5, 7, 9, 12, 15, 17 and 20), subscale 2—confusing/staring behaviour (items 3, 4, 6, 10, 13, 14, 19 and 21) and subscale 3—hostile behaviour (items 2, 8, 11, 16 and 18) [16].

The respondent is asked to rate how often s/he experiences stigmatization behaviours on a 5-point Likert scale: (1) never, (2) almost never, (3) sometimes, (4) often and (5) always. The total score is computed by adding all the item responses and dividing by the total number of items (items from subscale 1 are reverse coded because they are positively worded). Higher item scores always indicate greater perception of stigmatization behaviour. A Cronbach’s alpha value of 0.93 was obtained for the PSQ total score in the original study [4]. The final Brazilian version of the PSQ was developed following guidelines for the cross-cultural adaptation process: translation; obtaining the first consensus of Portuguese versions; evaluation by a panel of experts; back-translation; obtaining the consensus of versions in English and compared with the original English version; evaluation of the instrument by the original author; semantic analysis of the items and pre-test [17,18]. More details about the cross-cultural adaptation process of the PSQ into Brazilian Portuguese are available elsewhere [16].

#### Burns Specific Health Scale—Revised (BSHS-R)

The BSHS-R is a specific scale for assessing the health status of burned patients. It has 31 items divided into six domains: simple functional abilities, heat sensitivity, treatment regimens, affect and body image, work and interpersonal relationships. Scores range from 31 to 155, higher scores denoting better health status [20]. The BSHS-R has been adapted into Brazilian Portuguese and validated, with a Crobanch’s alpha value of 0.94 [21]. We applied the BSHS-R to assess the constructs body image and social relations using the domains affect and body image and interpersonal relationships, respectively.

#### Rosenberg Self-Esteem Scale (RSES)

We assessed self-esteem by administering the Brazilian Portuguese version of RSES [20]. This scale has 10 4-point Likert items, possible responses being (1) strongly agree, (2) agree, (3) disagree, (4) strongly disagree. Total scores range from 10 to 40 points, higher scores indicating more self-esteem [22]. RSES has been used in previous studies with Brazilian burn patients [21,23,24].

#### Beck Depression Inventory (BDI)

We applied the BDI Brazilian Portuguese version to assess depression. The BDI contains 21 items with four alternative degrees from 0 to 3 points, indicating the severity/persistence of depressive symptoms. The potential range of the BDI is 0 to 63, with higher scores reflecting more depressive symptoms [25]. The BDI has also been used in other studies with Brazilian burn patients [21,23].

#### Sociodemographic and clinical characteristics questionnaire

We developed a questionnaire to collect sociodemographic characteristics, such as gender, age, marital status, number of years of education, employment status and family monthly income. The clinical and burn characteristics collected were BSA, burn agent, number of surgical operations and scar visibility. To evaluate scar visibility the participants were asked: “When you are in public, do you think that your burn scars are visible to others?” (yes/no)

### Psychometric analyses

#### Measurement model

The factor structure (unidimensionality of subscales and second order factor structure for the global score) was evaluated by means of confirmatory factor analysis using ordinal categorical item indicators (CIFA) as follows: first, polychoric correlations were estimated based on a latent response threshold process; second, the factor analysis was conducted using a diagonally unweighted least squares estimation [26] on the polychorics [27,28]. Analyses were conducted using robust estimation as recommended by Moustaki and Victoria-Feser [29], to avoid the influence of multivariate non-normality, with corrections for robust p-values and standard errors to test the hypothesis of a three-factor PSQ model. Model fit was compared considering the absolute fit (chi-square [*χ*2] value using significance level over 0.05 for an acceptable model). Because the absolute fit was not achieved, we considered approximate fit using Root Mean Square Error of Approximation (RMSEA) ≤ 0.08; and relative fit using Comparative Fit Index (CFI) ≥ 0.95 and Tucker-Lewis Index (TLI) ≥ 0.95 [30], and overall residual considered acceptable if the Weighted Root Mean Residual (WRMR) < 0.95 [31, 32].

When a subscale did not show good fit to a unidimensional model, we refined the model by exploring item misfit to achieve subscale unidimensionality using a two-step approach:

1. We conducted Exploratory Factor Analyses of the misfitting subscale in a 50% random sample of individuals (training split), treating items as ordinal categorical and using unweighted least squares estimation. Adequacy of the EFA was tested if Bartlett’s test statistic [33] was significant with nominal level 0.05 and Kaiser-Meyer Olkin test of sampling adequacy (KMO) over 0.70 [34]. Test of common factor selection was conducted using Hull’s method [35], using the change in RMSEA as criterion. The item with highest residual correlation value on the single factor EFA solution was dropped from the analysis and the EFA was repeated until the unidimensional factor solution showed adequate fit
2. To avoid sample overfitting, this unidimensional subscale model was then tested as a confirmatory model in the remaining half of the sample (validation split), using the same criteria as above.

#### Reliability

Internal consistency was assessed by calculating Cronbach’s alpha for the PSQ total score and subscales, and for domains of BSHS-R, RSES and BDI [36]. Reproducibility of the PSQ was evaluated with a test-restest design and computed using as the two-way mixed effects intraclass correlation coefficient (ICC). For the Cronbach’s alpha and the ICC, values greater than 0.70 were considered acceptable [37].

The distribution of PSQ item responses was calculated in order to assess floor and ceiling effects. A floor or ceiling effect was considered to occur when more than 15% of the participants attained the lowest (floor) or highest (ceiling) possible score [38]. Item-total and item-subscale score correlations were analysed considering as adequate values between ≥ 0.20 and ≤0.70. Values below than 0.20 should be discarded [19].

#### Construct validity

Construct validity was assessed by the Multitrait Multimethod Matrix (MTMM) and known groups. The MTMM was constructed from the correlations between PSQ total and subscales, BSHS-R domains (affect and body image; interpersonal relationships), RSES and BDI. Pearson’s Correlation test was applied considering the following levels: below 0.30, weak correlation; between 0.30 and 0.50, moderate correlations; above 0.50, strong correlations [39].

Known-groups were defined according to scar visibility (yes vs no), burn severity (BSA < 20% vs ≥ 20%) and gender (female vs male). Mean PSQ total/subscales were compared among groups using the t-test for independent samples. Magnitude of the difference was measured by effect size (ES) coefficient (difference in mean scores between groups/pooled standard deviation). Effect sizes were classified as: no effect, below 0.20; small effect, between 0.20 and 0.50; moderate effect, between 0.51 to 0.80 and large effect, above 0.80, according to well-accepted standards [40]. Significance tests were all evaluated at the 0.05 level.

Confirmatory Factor Analyses were conducted with MPlus version 5.0 and Exploratory Factor Analyses was conducted with FACTOR [41]. All other analyses were done with SPSS version 20.0 for Windows.

## Results

A total of 329 burn patients were invited to participate in this study; five refused to participate and 84 did not meet the inclusion criteria. Finally, 240 Brazilian burn patients completed the interview. The mean age was 38.4 years (SD = 14.4) and the mean of years of education 8.8 years (SD = 3.9). Most participants were men (55%), had less than 20% of BSA (77.7%), had undergone at least one surgical operation for burns (79.2%), mean of 2.2 operations (SD = 3.1) and considered their scars visible to others (58.3%) (Table 1).

**Table 1:** Socioeconomic and clinical characteristics of the burn patients sample (n = 240).

Variables	N	(%)	Total mean PSQ	(SD)
**Socioeconomic variables**				
**Gender**				
Male	132	(55)	1.9	(0.7)
Female	108	(45)	1.9	(0.6)
**Age in years**, mean (SD^a^)	38.4	(14.4)	N.A	N.A.
**Clinical variables**				
**Hospitalized**	187	(77.9)	2.0	(0.6)
**BSA**^b^, mean (SD)—missing data 16	12.8	(13.6)	N.A	N.A.
< 20%	174	(77.7)	1.9	(0.6)
≥ 20%	50	(22.3)	2.1	(0.6)
**Burning agentes**				
Scald	52	(21.7)	1.9	(0.6)
Alcohol on fire	44	(18.3)	2.0	(0.6)
Contact / Abrasion	44	(18.3)	1.9	(0.6)
Direct fire	34	(14.2)	1.9	(0.7)
Others (Electricity, Explosion, Freezing, Chemical)	66	(27.5)	2.1	(0.6)
**Number of patients undergoing surgery**—missing data 8	190	(79.2)	2.0	(0.6)
**Number of operations per patien**t mean (SD), missing data 31	2.2	(3.1)	N.A	N.A.
**Scar visibility**, yes	140	(58.3)	2.1	(0.6)

N.A. = not applicable

^a^ Standard deviation

^b^ Body surface area burned

### Confirmatory Item Factor Analysis (CIFA)

Table 2 shows fit values for the first-order subscales. As may be seen there, goodness-of-fit indices were satisfactory for subscales 2 and 3, but unacceptable for subscale 1 (*χ*2 = 185.90; df = 20; RMSEA = 0.11; CFI = 0.93; TLI = 0.87; WRMR = 1.13).

**Table 2:** Goodness-of-fit indices for a three-factor model of the BR-PSQ-R subscales.

Subscales	df^a^	*χ*2^b^(*p-value*)	RMSEA^c^	CFI^d^	TLI^e^	WRMR^f^
PSQ1: absence of friendly behavior	20	85.90 (<0.01)	0.11	0.93	0.87	1.13
PSQ1-R: absence of friendly behavior (validation sample)	5	10.10 (0.10)	0.09	0.98	0.97	0.86
PSQ2: confusing/staring behavior	20	27.45(0.12)	0.04	0.99	0.99	0.55
PSQ3: hostile behavior	25	7.02 (0.22)	0.04	0.99	0.99	0.34

^a^df: degrees of freedom;

^b^ *χ*2: chi-square;

^c^RMSEA: Root Mean Square Error of Approximation;

^d^CFI: Comparative Fit Index;

^e^TLI: Tucker-Lewis Index;

^f^ Weighted Root Mean Residual

Thus, we proceeded to test an alternative solution in half of the sample, chosen at random (“training sample”, n = 120) using EFA. Sampling adequacy was considered satisfactory, given that Bartlett’s test for EFA was significant (*χ*2 = 225.7; df = 10; *p*<0.001) and KMO value adequate (0.73). During EFA iterations, items 1, 12 and 17 were dropped due to large average residual correlation (0.38, 0.22 and 0.26, respectively). After dropping items, this refined version of the PSQ-1 subscale (PSQ1-R) presented a satisfactory one-dimensional structure with adequate goodness-of-fit indices in the training sample (*χ*2 = 10.10; df = 5; *p* = 0.10; RMSEA = 0.09; CFI = 0.98; TLI = 0.97; WRMR = 0.86). The validation sample indices were also adequate in terms of absolute (*χ*2 = 10.10; df = 5; *p* = 0.10), and relative fit (CFI = 0.98; TLI = 0.97).

**Fig 1:**
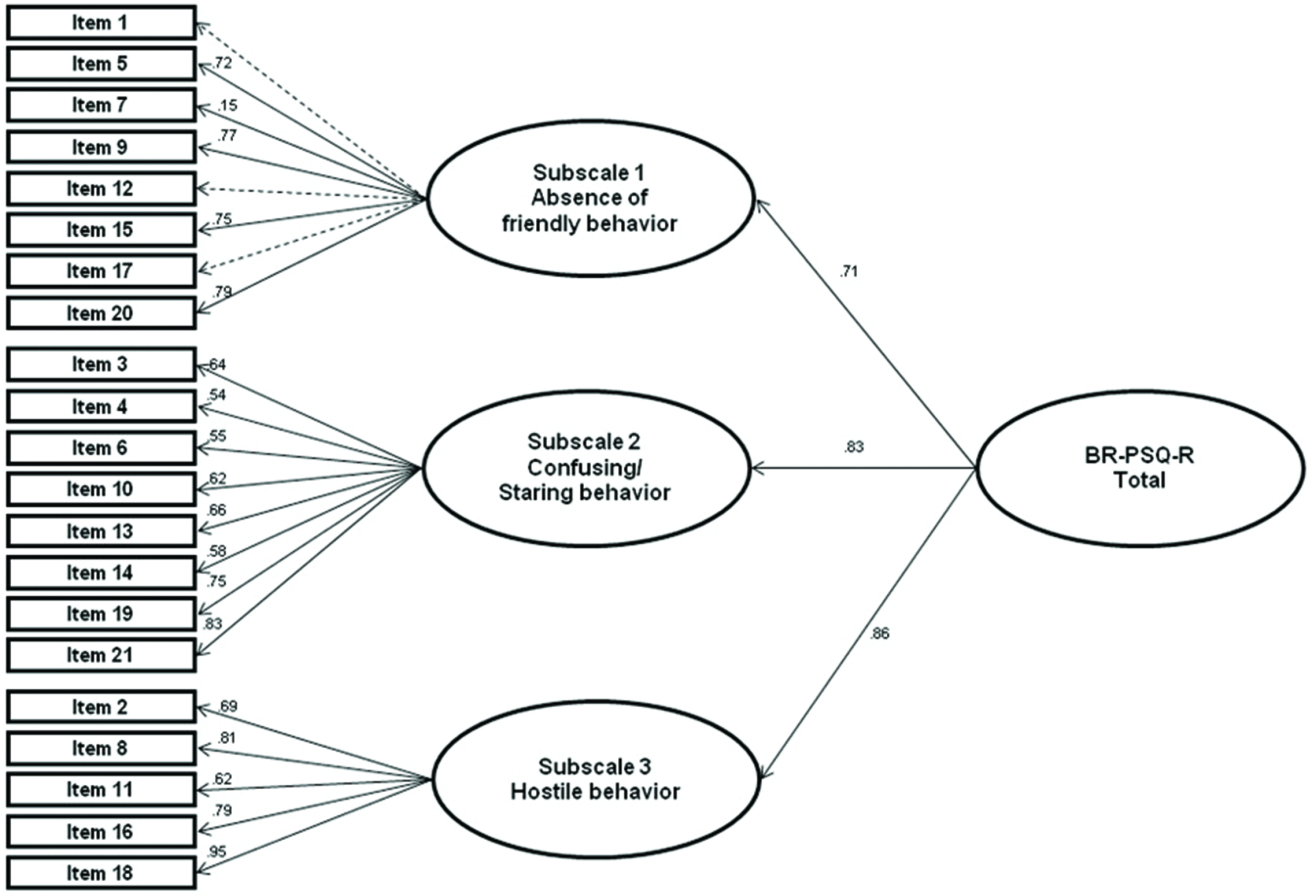
Path diagram of the CIFA measurement model of the BR-PSQ-R total score. df: degrees of freedom; *χ*2: chi-square; RMSEA: Root Mean Square Error of Approximation; CFI: Comparative Fit Index; TLI: Tucker-Lewis Index.

When the full structure was tested in the overall sample using the original version, the fit of the second order hierarchical model was satisfactory (*χ*2 = 338.40; df = 186; RMSEA = 0.06; CFI = 0.92; TLI = 0.96; WRMR = 0.95). When the refined version of subscale 1 was tested, the model showed similar fit values (*χ*2 = 258.23; df = 132; *p*<0.01; RMSEA = 0.06; CFI = 0.94; TLI = 0.94; WRMR = 0.95), indicating that the overall score was an adequate summary of the 3 subscales in both cases. Fig 1 presents the three-factor hierarchical model, the dashed lines apply to the items 1, 12 and 17 that were dropped due to large average residual correlation.

### Reliability

As shown in Table 3 all items of the refined version of BR-PSQ (BR-PSQ-R, S1 Appendix) presented floor effects. However, no ceiling or floor effects were observed for the BR-PSQ total score. Only BR-PSQ-R subscale 3, “hostile behaviour”, showed floor effects of over 40%. Item-total score correlation and item-subscale score correlations ranged from 0.02 to 0.67 and 0.19 to 0.69, respectively (Table 3). Internal consistency of the BR-PSQ-R total and subscales was high, with Cronbach’s alpha values ranging from 0.65 to 0.88. Cronbach’s alphas for the BSHS-R subscales, BDI and RSES ranged from 0.85 to 0.93 (Table 4).

**Table 3:** BR-PSQ-R item floor and ceiling effects, item-total score correlation and item-subscale score correlation.

BR-PSQ-R Subscales	Items	1	2	3	4	5	Floor Effect %	Ceiling Effect %	Missing Items %	Item-total score correlation	Item-subscale score correlation
**Subscale 1** Absence of friendly behaviour (reversed items)	1	--	--	--	--	--	--	--	--	--	--
	5	2.5	1.7	17.9	20.4	57.5	57.5	2.5	0.0	0.45	0.44
	7	11.7	5.9	36.0	9.2	37.2	37.2	11.7	0.4	0.02	0.19
	9	2.9	3.3	21.3	17.6	54.8	54.8	2.9	0.4	0.48	0.46
	12	--	--	--	--	--	--	--	--	--	--
	15	0.8	2.5	17.9	20.8	57.9	57.9	0.80	0.0	0.45	0.54
	17	--	--	--	--	--	--	--	--	--	--
	20	0.4	1.7	12.5	13.8	71.7	71.7	0.4	0.0	0.44	0.54
	Total	--	--	--	--	--	10.5	0.0	0.4	--	--
**Subscale 2** Confusing/Staring behaviour	3	69.2	7.9	17.5	2.9	2.5	69.2	2.5	0.0	0.45	0.40
	4	35.6	7.5	27.6	10.9	18.4	35.6	18.4	0.4	0.45	0.58
	6	27.6	7.1	43.5	11.3	10.5	27.6	10.5	0.4	0.44	0.51
	10	54.0	6.3	23.4	4.6	11.7	54.0	11.7	0.4	0.47	0.45
	13	50.0	9.6	25.8	5.8	8.8	50.0	8.8	0.0	0.49	0.52
	14	31.4	7.9	31.0	9.6	20.1	31.4	20.1	0.4	0.45	0.49
	19	39.6	5.4	30.0	8.8	16.3	39.6	16.3	0.0	0.57	0.52
	21	61.9	8.8	16.7	4.2	8.4	61.9	8.4	0.4	0.62	0.59
	Total	--	--	--	--	--	8.9	0.4	1.7	--	--
**Subscale 3** Hostile behaviour	2	75.0	8.8	10.8	3.3	2.1	75.0	2.1	0.0	0.45	0.51
	8	70.8	7.5	9.2	5.0	7.5	70.8	7.5	0.0	0.55	0.60
	11	56.7	4.6	21.3	6.7	10.8	56.7	10.8	0.0	0.42	0.47
	16	86.7	2.9	5.0	2.9	2.5	86.7	2.5	0.0	0.49	0.55
	18	73.3	5.8	12.9	3.8	4.2	73.3	4.2	0.0	0.67	0.69
	Total	--	--	--	--	--	40.0	0.4	0.0	--	--
**BR-PSQ Total**		--	--	--	--	--	1.7	0.0	1.7	--	--

**Table 4:** Multitrait-multimethod matrix of the BR-PSQ-R with self-esteem (RSES), depression (BDI), affect/body image and interpersonal relationships (BSHS-R). Cronbach’s alpha coefficients shown in the diagonal (in brackets).

	**BR-PSQ-R total score**	**BR-PSQ-R Subscale 1**	**BR-PSQ-R Subscale 2**	**BR-PSQ-R Subscale 3**	**RSES**	**BDI**	**Affect/body image**	**Interpersonal relationship**
BR-PSQ-R total score	[.88]							
BR-PSQ-R subscale 1	.**63**	[.65]						
BR-PSQ-R subscale 2	.**90**	.37	[.80]					
BR-PSQ-R subscale 3	.**78**	.36	.53	[.78]				
RSES	-.**57**	-.29	-.55	-.43	[.85]			
BDI	.**63**	.30	.57	.56	-.66	[.93]		
BSHS-R subscales: Affect/body image	-.**63**	-.35	-.63	-.41	.63	-.69	[.89]	
BSHS-R subscales: Interpersonal relationships	-.**55**	-.26	-.51	-.46	.55	-.71	.70	[.81]
Mean (SD) ^a^	2.0 (0.6)	1.8 (0.7)	2.3 (0.9)	1.6 (0.8)	21.7 (5.3)	12.3 (12.2)	30.2 (9.1)	21.4 (4.9)

^a^ Mean scores and standard deviation (SD) of the subscales

All Pearson’s correlations were significant at *p-value* < 0.001

Strength of correlations are marked in bold according to hypotheses of the original PSQ study

The test-retest sample was similar to the overall study sample: 50% were men, mean age of 41.5 years old (SD = 14.3), 8 years of education (SD = 3.0) and BSA mean of 16.2 (SD = 9.8). The mean time elapsed between the first and second interviews was 2.7 weeks (SD = 0.69). Test-retest reliability, as measured with ICC, was 0.87 for BR-PSQ-R total score; 0.82 for subscale 1; 0.87 for subscale 2 and 0.79 for subscale 3.

### Construct validity

**Fig 2:**
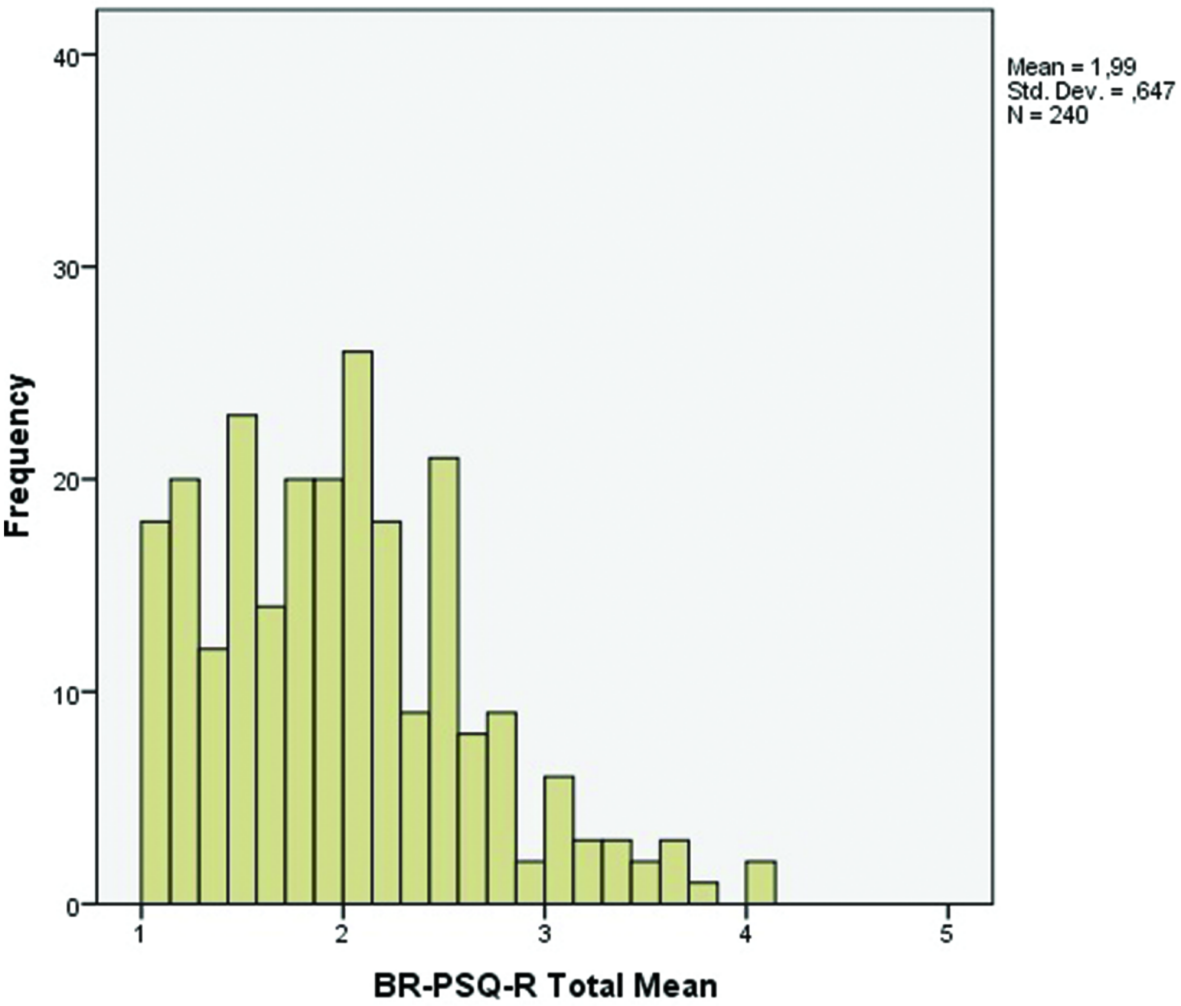
Distribution of responses of BR-PSQ-R total score.

Consistent with our hypotheses, Table 4 shows that the BR-PSQ-R total and subscale scores had strong positive correlations with BDI, and strong negative correlations with RSES and BSHS-R domains (affect and body image and interpersonal relationships). All correlations were statistically significant (*p* < 0.001). Mean BR-PSQ-R total score was 2.0 (SD = 0.6) and subscales mean scores ranged from 1.6 (SD = 0.8) to 2.3 (SD = 0.9), with higher mean for subscale 2 (Confusing/staring behavior) (Table 4, Fig 2).

Analysis of known-groups revealed statistically significant for scar visibility for the BR-PSQ-R total score (*p* <0.01), subscale 1 (*p* = 0.03) and subscale 2 (*p* = <0.01). Score effect sizes were large for scar visibility in Total score and Subscale 2 (0.54 and 0.58, respectively), and low in subscales 1 and 3 (0.15 and 0.26). In relation to the BSA and gender, there were no statistically significant differences in BR-PSQ-R scores (Table 5).

**Table 5:** Known-group differences in BR-PSQ-R total and subscales scores.

	*n* (%)	BR-PSQ total score Mean (SD)	Subscale 1 Mean (SD)	Subscale 2 Mean (SD)	Subscale 3 Mean (SD)
**Scar visibility**					
Yes	127	2.1 (0.6)	1.9 (0.7)	2.5 (0.9)	1.7 (0.9)
No	93	1.8 (0.5)	1.8 (0.6)	2.0 (0.8)	1.5 (0.7)
T(df = 218) p-value ^a^		3.33 <0.01	2.22 0.03	4.26 <0.01	1.71 0.08
Effect size		0.54	0.15	0.58	0.26
**BSA** ^b^					
≥ 20%	49	2.1 (0.6)	1.8 (0.6)	2.5 (0.9)	1.7 (0.9)
< 20%	171	1.9 (0.6)	2.0 (0.7)	2.3 (0.8)	1.6 (0.8)
T(df = 218) p-value ^a^		0.79 0.09	0.53 0.14	1.71 0.14	3.34 0.45
Effect size		0.33	0.14	0.24	0.11
**Gender**					
Female	97	2.0 (0.6)	1.8 (0.7)	2.4 (0.9)	1.7 (0.9)
Male	123	2.0 (0.5)	1.9 (0.6)	2.3 (0.8)	1.6 (0.8)
T(df = 218) p-value ^a^		3.46 0.82	0.07 0.80	1.70 0.65	0.018 0.99
Effect size		0.00	0.00	0.00	0.00

^a^ t-test for independent samples;

^b^ Body surface area burned

**Fig 3:**
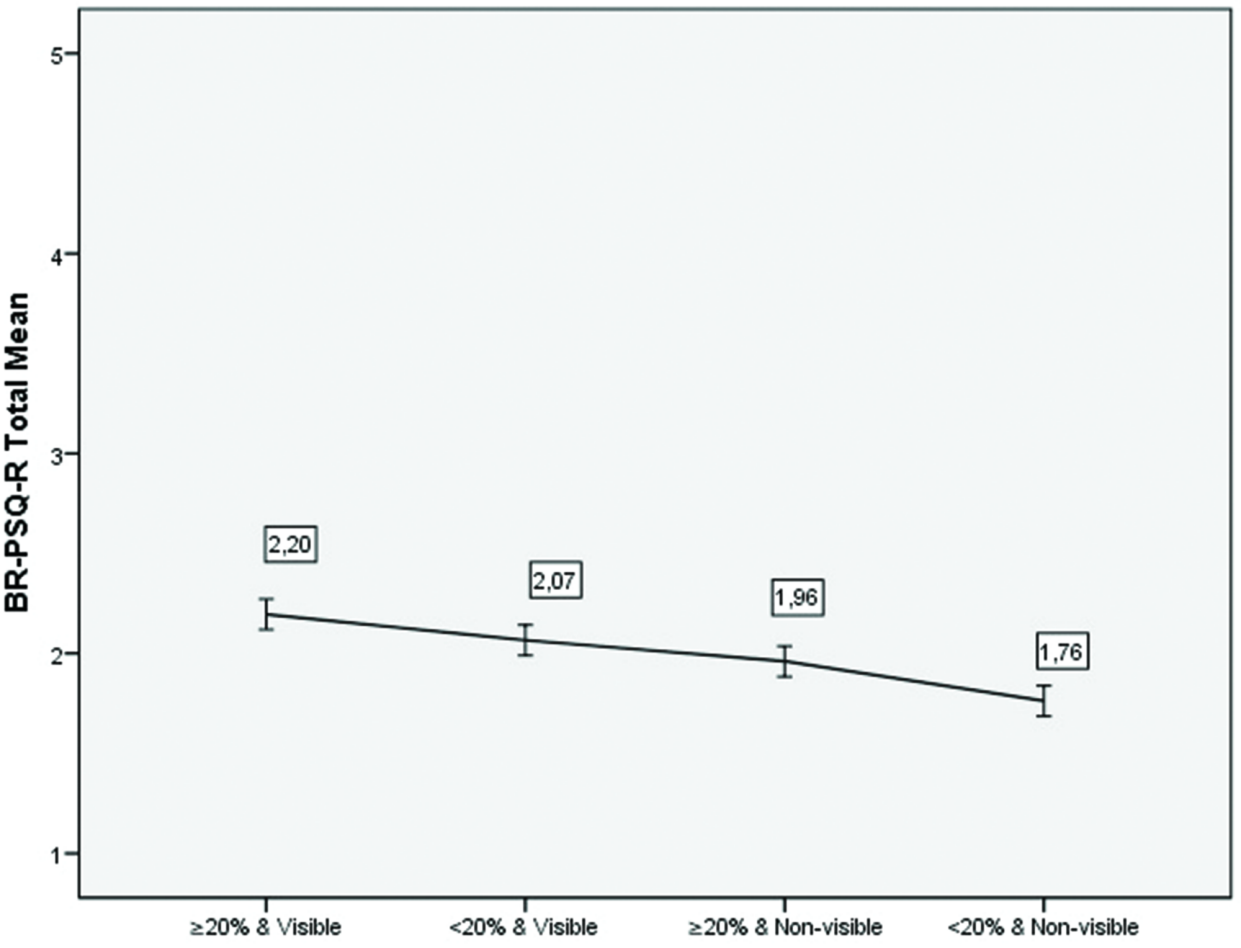
Mean of the BR-PSQ-R total score across BSA and scar visibility.

We explored whether there was a severity gradient given in the BR-PSQ-R in terms of combination of BSA and between scar visibility. We defined 4 groups of theoretical stigma, from high to low: ≥ 20% BSA & Visible scar; <20% BSA and visible scar; ≥20% BSA and non-visible scar, and <20% and non-visible scar. Using a 4-group ANOVA, we found a linear negative association between BR-PSQ-R scores and theoretical stigma, (F = 12, df1 = 1, df2 = 220, *p* = 0,001). Thus, results indicate that higher total BR-PSQ-R scores indicate higher stigma for patients with higher BSA and also for patients who considered that their scars were visible to others(Fig 3).

## Discussion

The results of this study showed that the BR-PSQ-R is a valid instrument to assess perceived stigmatization among Brazilian burn patients. This is the first instrument available that assesses this construct among the Brazilian Portuguese population.

In this study, we found that the measurement model of the BR-PSQ was better explained in terms of first-order subscales when some items were dropped from the subscale 1. However, regarding the full structure, both versions of the BR-PSQ showed goodness-of-fit, validity and reliability indices which were similar to the original version [4], confirming our previous hypotheses. The total score of the BR-PSQ-R showed slightly better fit values according to the RMSEA than the original version. The absence of friendly behaviour trait showed items with low factor loadings. This result agrees with tests performed with the original version, that showed that the goodness-of-fit indices of this subscale had a worse fit (n = 361; RMSEA = 0.11; CFI = 0.80; TLI = 0.76) [4]. Thus, after dropping items 1, 12 and 17 from subscale 1 for the training sample, we obtained better goodness-of-fit indices.

We found lower values of the item-correlation and factor loading of the item 7 that had been already reported for the original version of PSQ when tested in child burns [14]. This finding suggests that the BR-PSQ can be better interpreted in terms of its total score than in terms of its subscale scores, as in the original version. More results are needed in cross-cultural comparisons, and Differential Item Functioning (DIF) analyses are needed to study the robustness of the PSQ measurement model across cultures and languages [42,43]. In addition to cross-cultural issues, gender and education are also important [44].

All items of the BR-PSQ showed some floor effects. The presence of a floor effect reflects low discriminatory power [45], even though the instrument was found to be reliable. It indicates that the instrument may not be sensitive enough to assess low levels of perceived stigmatization [38]. The fact that most of the participants of our study had a low educational level may explain this result.

In terms of reliability, the Cronbach’s alpha coefficients were above the standard threshold [37] and only marginally lower than those of the original version [4]. We have also found good values for the ICC in test-retest reliability and, again, above the standard threshold [37]. The latter result represents incremental evidence about the PSQ, as—to our knowledge—there is no evidence of the temporal stability of the American version.

We had hypothesized that patients with a higher BSA and scar visibility would have a greater perception of stigmatization behaviour. As in the US sample of adults [4], we found a small but significant association between the PSQ and both BSA and scar visibility. We also compared PSQ scores of men and women patients. Some studies had reported an association between female gender and poorer body image among burn survivors [46], but this finding has not been consistent [47]. In our study, we found no association between gender and perceived stigmatization as measured by the BR-PSQ-R.

As hypothesized, we found that the BR-PSQ-R scores showed strong positive correlations with BDI (depression), and strong negative correlations with RSES (self-esteem) and with BSHS-R subscales (affect/body image and interpersonal relationships). Other studies [48, 4] found similar results. Reporting perceived stigmatizing behaviours is associated to the inability to have a social life, to form and maintain friendships and gain peer acceptance, important aspects of social reintegration. According to another study, perceived lack of friendly social interactions may be linked to depression [48].

This study has some limitations that deserve further comment. First, the sample size did not allow conducting certain clinically relevant comparisons, for instance BR-PSQ-R score depending on the number of surgical operations. However, we were able to recruit all eligible patients from two large burns units in Brasil, allowing us to achieve a sufficiently large sample size to comply with recommendations from consensus guidelines in conducting our main comparisons [19]. Second, our study has limited comparability with the original US study, due to differences between the samples. In the US study, participants had larger burns (mean BSA = 47.7%) [4] than in our sample (mean BSA = 12.8%). Despite this difference in burn severity between the samples, the factor structure of the PSQ was very similar in both samples. Further research should address the invariance of the PSQ model comparing the structure of the US and Brazilian versions, so that cultural aspects of stigma can be addressed. A third limitation is the cross-sectional design of our study, which did not allow us to assess the scale responsiveness. More research is still needed in order to obtain clearer evidence of the sensitivity to change of the BR-PSQ-R.

On the other hand, the current study makes several contributions to the literature. Our sample was naturalistic with high external validity: we included all patients in two burns units with a broad range of clinical characteristics. The BR-PSQ-R factor structure, and reliability and validity indices were very similar to those found among both adult and children burn survivors in the US [4]. Moreover, this study is the first to investigate test-retest reliability of the PSQ.

## Conclusion

Our results provide evidence that the BR-PSQ-R is an adequate instrument to assess the perceived stigmatization commonly reported by adult burns survivors, similar to the original American version.

## Supporting information

S1 Appendix Brazilian refined version of the *Perceived Stigmatization Questionnaire* (BR-PSQ-R). (PDF)
