# Supplementary material for: Validation of the Perceived Stigmatization Questionnaire for Brazilian adult burn patients
Source: PLoS One. 2018 Jan 30;13(1):e0190747. doi: 10.1371/journal.pone.0190747 (PMC5790232; doi:10.1371/journal.pone.0190747)
Supplement: S1 Appendix — (PDF) [file pone.0190747.s001.pdf]

Appendix 1

Brazilian refined version of the *Perceived Stigmatization Questionnaire* (BR-PSQ-R)

Versão Brasileira Refinada do *Perceived Stigmatization Questionnaire* (BR-PSQ-R)

<u>Como as outras pessoas tratam você?</u>

Durante seu dia a dia, você provavelmente se encontra e conversa com muitas pessoas diferentes. Gostaríamos de saber com que frequência as pessoas agem de determinadas formas em relação a você.

Para cada pergunta, indique a frequência com a qual as pessoas fazem certas coisas. Avalie como as pessoas trataram você durante o último ano.

Use a escala abaixo, *nunca* (1) a *sempre* (5). Assinale suas respostas com um círculo.

Nunca<br>1	Quase Nunca<br>2	Às Vezes<br>3	Muitas vezes<br>4	Sempre<br>5

1. As pessoas me xingam.	1	2	3	4	5
2. As pessoas evitam olhar para mim.	1	2	3	4	5
3. Pessoas que não conheço ficam surpresas ou assustadas quando me veem.	1	2	3	4	5
4. As pessoas são gentis comigo.	1	2	3	4	5
5. As pessoas não sabem o que me dizer.	1	2	3	4	5
6. Pessoas que não conheço dizem “Oi” para mim.	1	2	3	4	5
7. As pessoas riem de mim.	1	2	3	4	5
8. As pessoas ficam à vontade na minha presença.	1	2	3	4	5

9. As pessoas têm pena de mim.	1	2	3	4	5
10. As pessoas implicam comigo (pegam no meu pé).	1	2	3	4	5
11. As pessoas não sabem como agir na minha presença.	1	2	3	4	5
12. As pessoas me olham e se viram para me olhar.	1	2	3	4	5
13. As pessoas são simpáticas comigo.	1	2	3	4	5
14. As pessoas me fazem ameaças (*bulling*).	1	2	3	4	5
15. As pessoas debocham de mim.	1	2	3	4	5
16. Pessoas que não conheço me encaram.	1	2	3	4	5
17. As pessoas me tratam com respeito.	1	2	3	4	5
18. As pessoas parecem desconfortáveis com minha aparência.	1	2	3	4	5

Instruções de Avaliação do QEP:

Perguntas de código reverso 5, 7, 9, 15, 20

Pontuação da Subescala para Ausência de Comportamento Amigável: (1+5+9+15+20)/5

Pontuação da Subescala para Comportamento Confuso / Olhar Fixo: (3+4+6+10+13+14+19+21)/8

Pontuação da Subescala para Comportamento Hostil: (2+8+11+16+18)/5

Resultado Total PSQ: Some todos os itens e divida por 18

Os resultados da escala são calculados somando-se os itens e dividindo-se pelo número de itens da escala. Portanto, os resultados da escala são médias e se encontram na mesma métrica da escala de frequência de 5 pontos para que as pontuações da escala

sejam comparáveis e facilmente interpretadas. Contagens elevadas indicam níveis altos de comportamento de estigmatização percebida.

Noélle O. Freitas (2015): translated with kind permission of John W. Lawrence, PhD.
